# Supplementary material for: Embryonic liver developmental trajectory revealed by single-cell RNA sequencing in the Foxa2eGFP mouse
Source: Commun Biol. 2020 Nov 3;3:642. doi: 10.1038/s42003-020-01364-8 (PMC7642341; doi:10.1038/s42003-020-01364-8)
Supplement: Supplementary file 1 — Supplementary Information [file 42003_2020_1364_MOESM1_ESM.pdf]

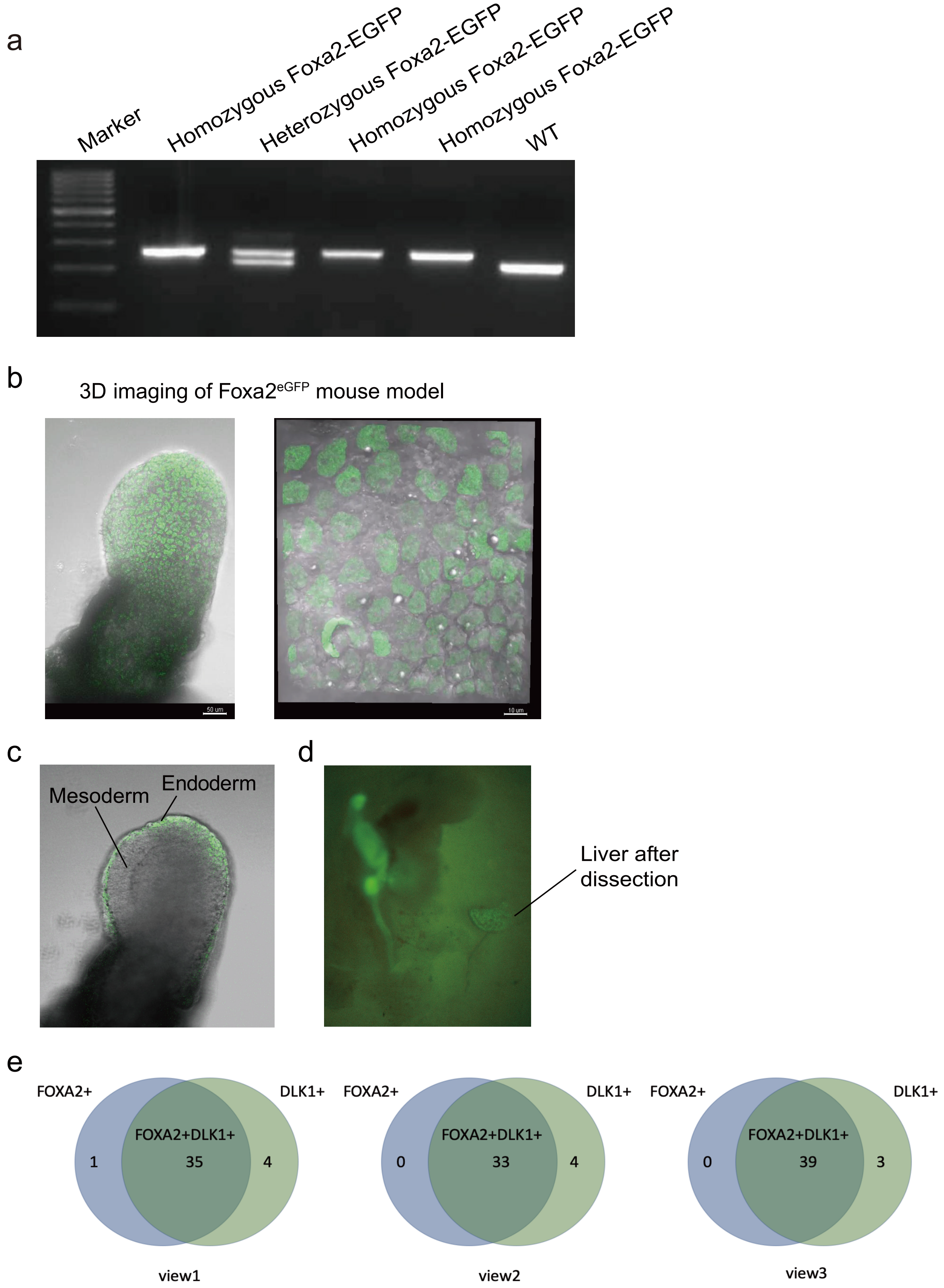

Supplementary Figure 1 | The transgenic Foxa2^{eGFP} reporter mouse model. a Genotyping of homozygous Foxa2^{eGFP} (269 bp), heterozygous Foxa2^{eGFP} (230/269 bp) and wild type (230 bp) mice was shown. **b** 3D mouse imaging showed 6hs embryo culture of Foxa2^{eGFP} at E6.5. **c** Endoderm and mesoderm of Foxa2^{eGFP} mouse model. Scale bar, 50 μm for left panel 10 μm for right panel. **d** Demonstration of microdissection of liver at E11.5. **e** Quantification of co-labeling immunofluorescence assay of FOXA2 and DLK1 of three replicates at E12.5 by venn plot.

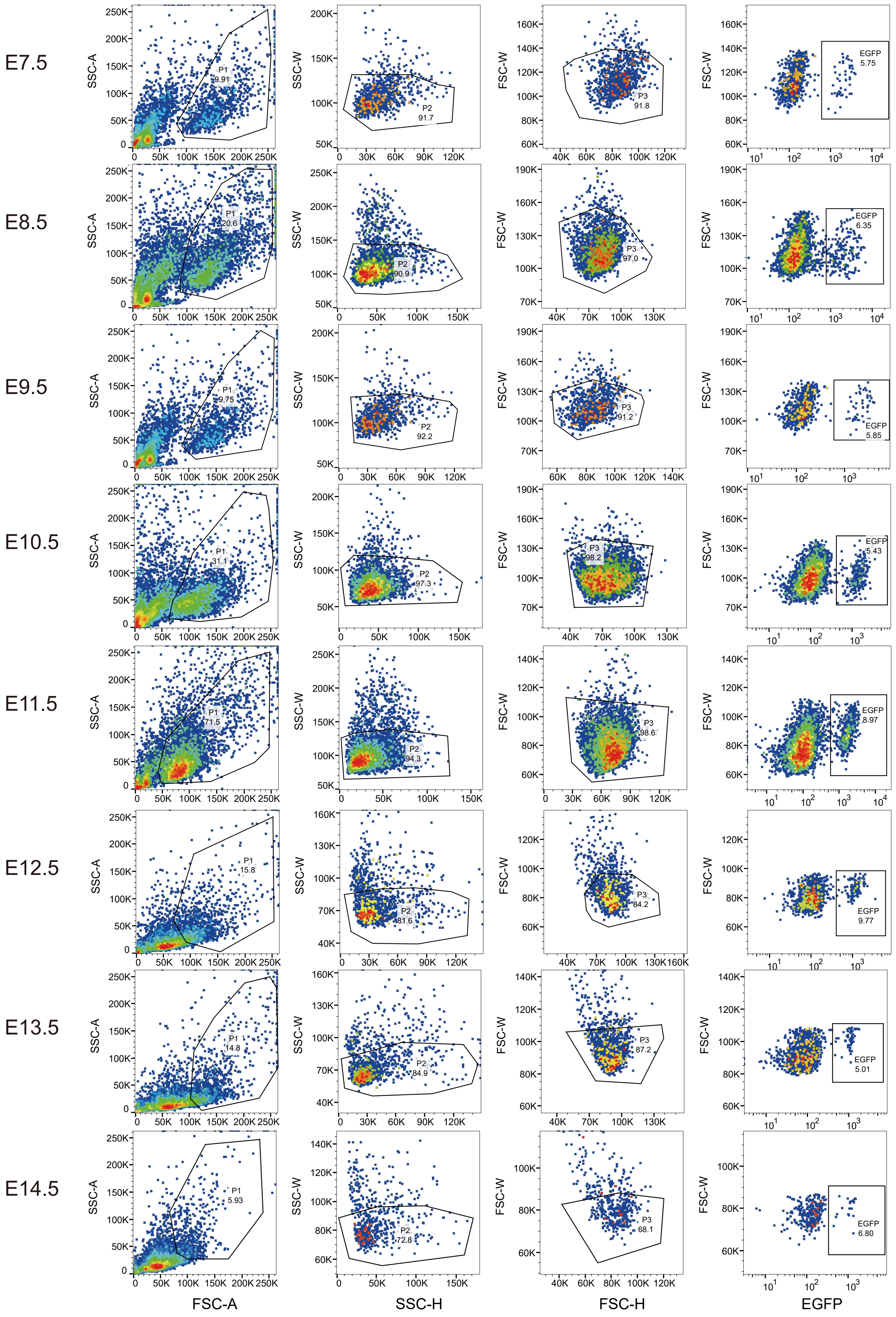

Supplementary Figure 2 | FACS sorting parameters of Foxa2^{eGFP} mice by eGFP during E7.5-E14.5. Doublets and multiplets were excluded by gating of side scatter (SSC) and forward scatter (FSC).

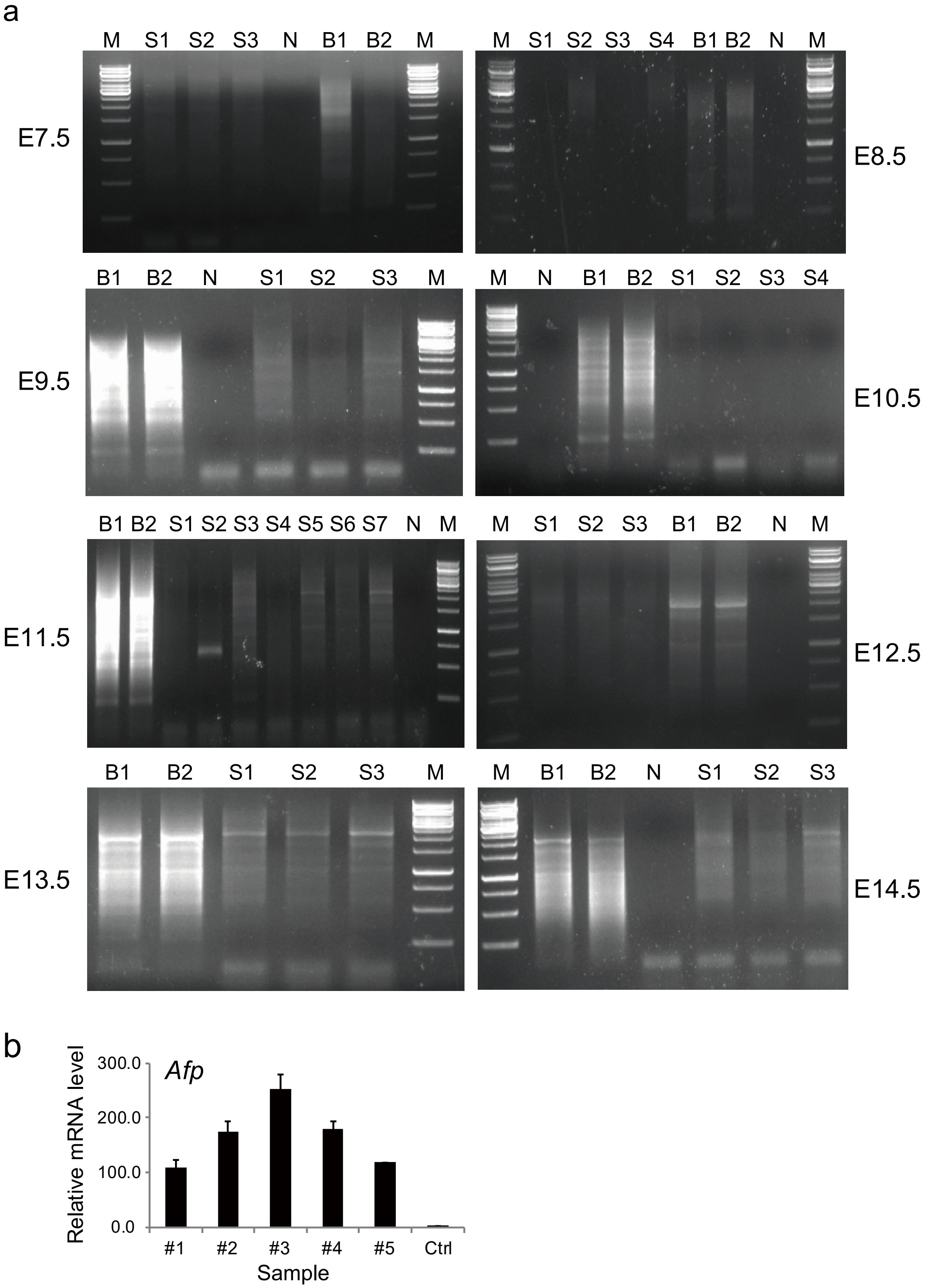

Supplementary Figure 3 | Assessment of the amplified cDNA and mRNA. a The amplified cDNA was assessed by agarose gel. **b** The mRNA level of *Afp*, a hepatic marker, was assessed by qPCR, before library generation to ensure the sequencing quality. M, Marker; S1-S7, Single cell 1-7; N, negative control. B1-B2, bulk 1-2.

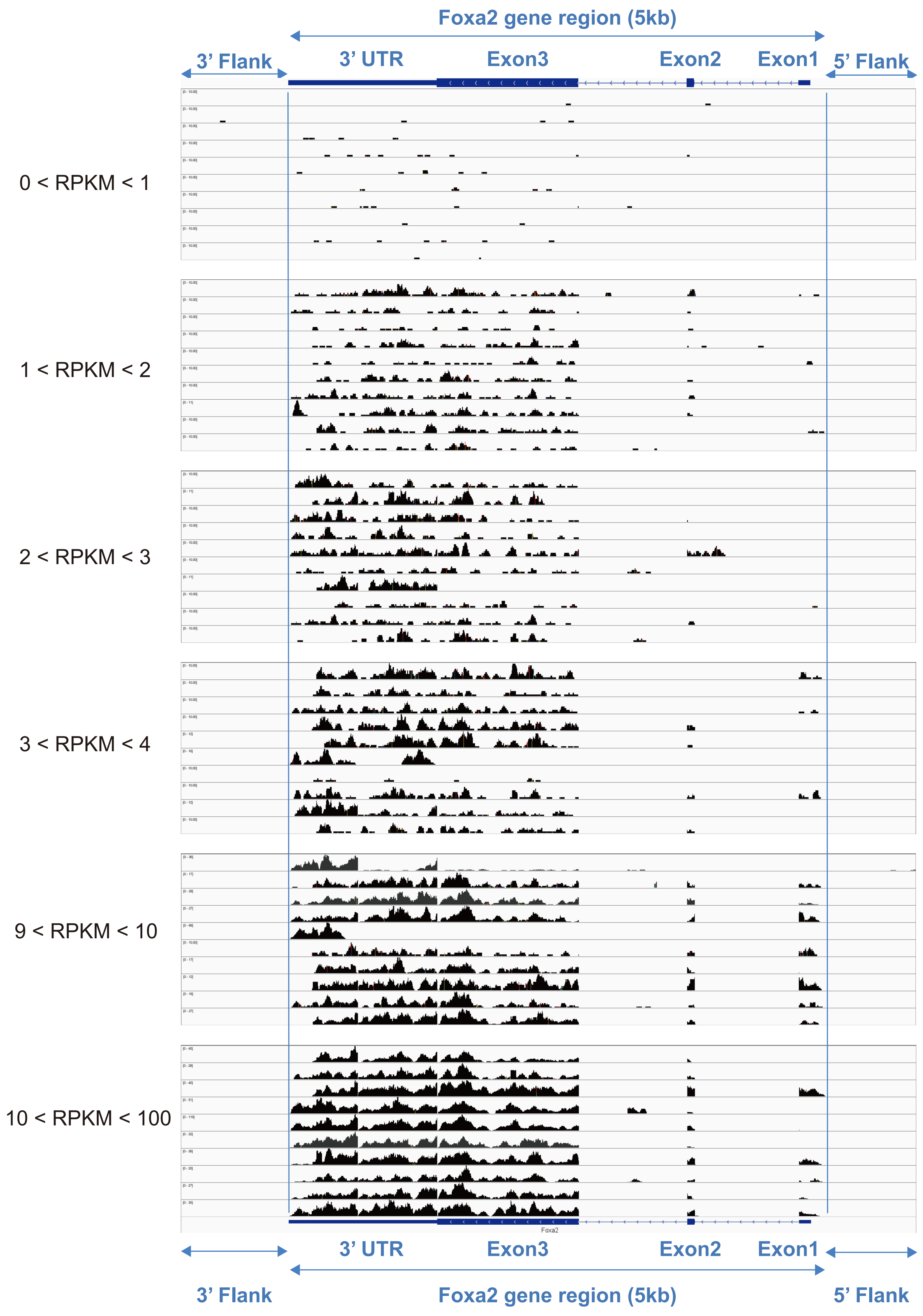

Supplementary Figure 4 | Determination of RPKM threshold for gene detection. The gene expression level was characterized by RPKM (Reads Per Kilobase per Million mapped reads). The reads coverage of the *Foxa2* gene in the randomly selected 10 cells with different RPKM (0<RPKM<1, 1<RPKM<2, 2<RPKM<3, 3<RPKM<4, 9<RPKM<10, 10<RPKM<100) was shown. Based on the reads coverage pattern, RPKM>1 was used as the threshold for gene expression.

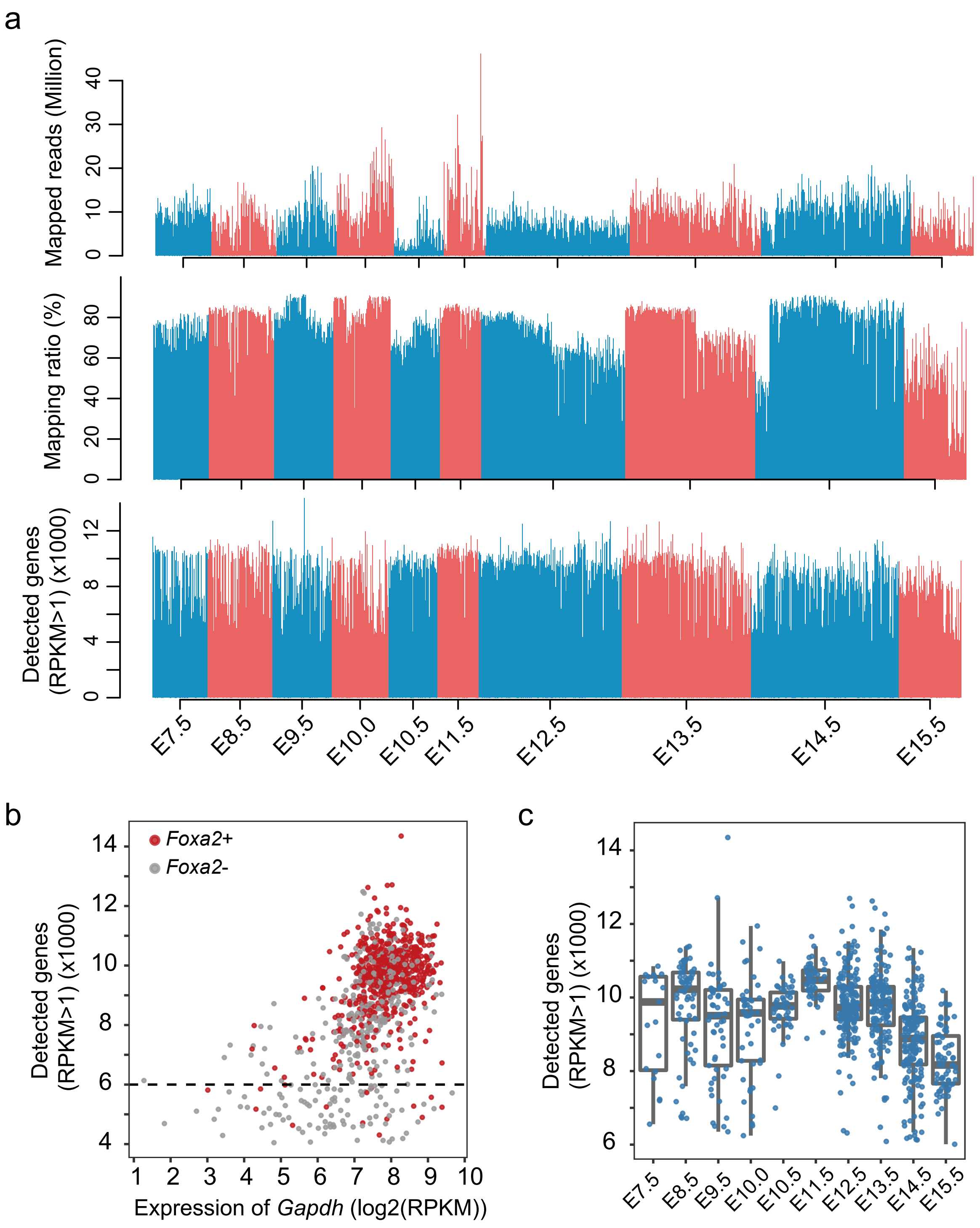

Supplementary Figure 5 | Statistics of single-cell RNA-seq. a Statistics of the number of mapping reads, mapping ratio and detected genes of each single cell were shown. **b** Dot plot demonstrated the detected gene numbers and expression level of *Gapdh* in each single cell. Cells were filtered if the detected genes were less than 6,000. **c** Box plot of the detected genes of each single cell from E7.5 to E15.5 after filtering.

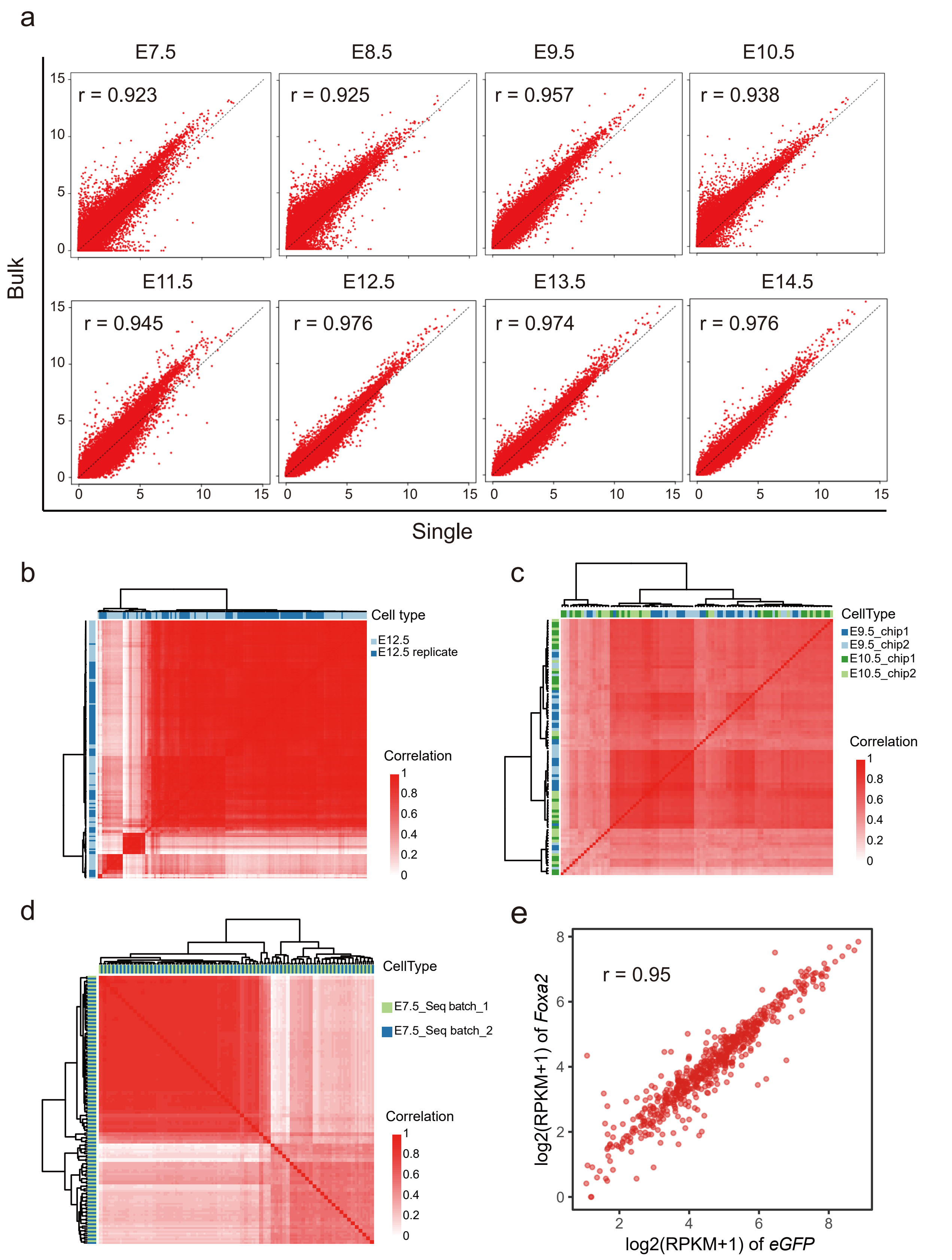

Supplementary Figure 6 | Quality control of single-cell RNA-seq. a Correlation of average gene expression between single cells and bulk samples. **b-d** Technical noise was assessed by calculating the Pearson correlation between experimental replicates (**b**), chips pooled different embryonic day (**c**) and sequencing batches (**d**). **e** As *eGFP* and *Foxa2* were co-expressed in our mouse model, a high correlation was detected (r = 0.95) between these two genes.

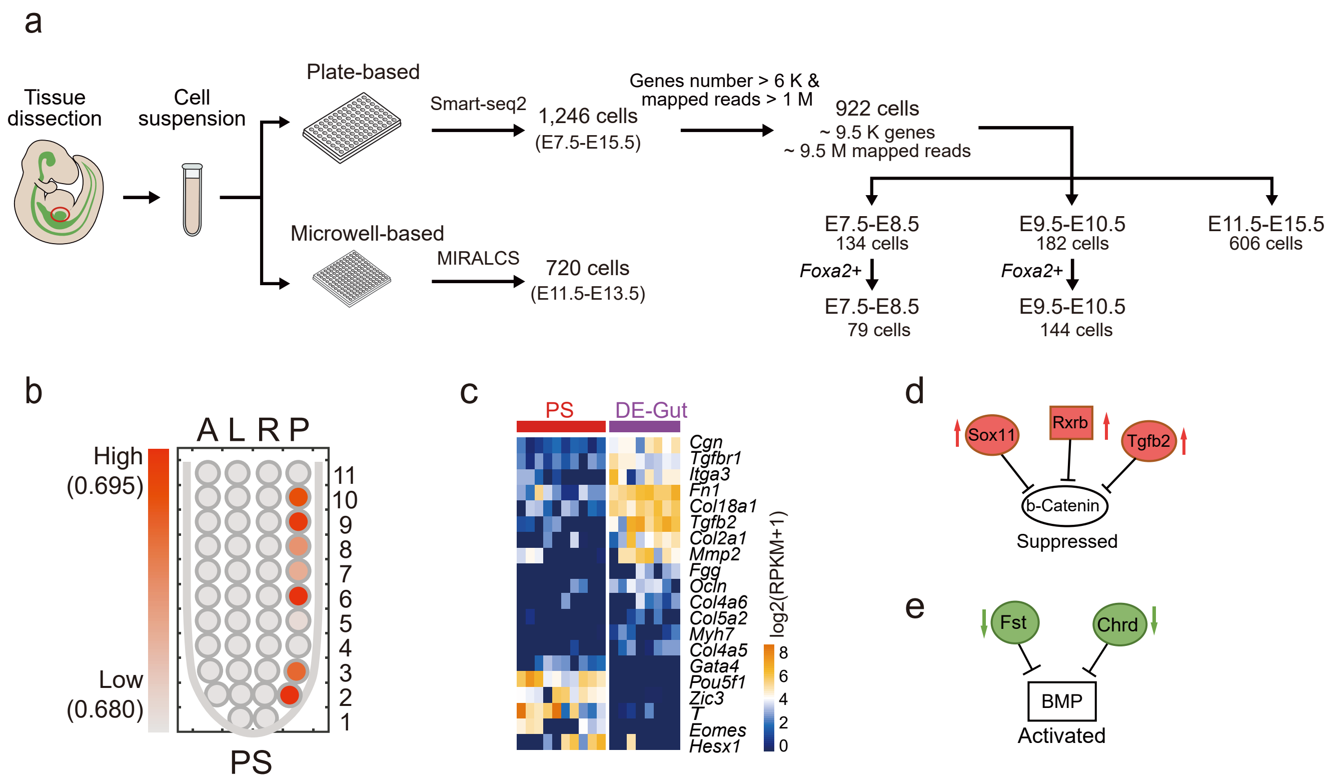

Supplementary Figure 7 | Mapping the endoderm development. a Graphical depiction of different parameters at each developmental time-point. The parameters, including minimal expressed gene number per cell, minimal mapped reads per cell, cell sub-clusters (*Foxa2*+/-) and cell number for each cluster at different developmental time-points are shown. **b** Primitive streak cells were mapped to the iTranscriptome database. The color indicated the gene expression level. The locations of the cells were defined by A (Anterior), P (Posterior), L (Left), and R (Right). **c** Heatmap illustrated the part of genes that differentially expressed between the PS and DE-Gut. **d** The Wnt/β-catenin signaling pathway was identified to be repressed by *Sox11*, *Rarb* and *Tgfb2* in DE-Gut. **e** BMP signaling was activated by repressing *Fst* and *Chrd* in DE-Gut.

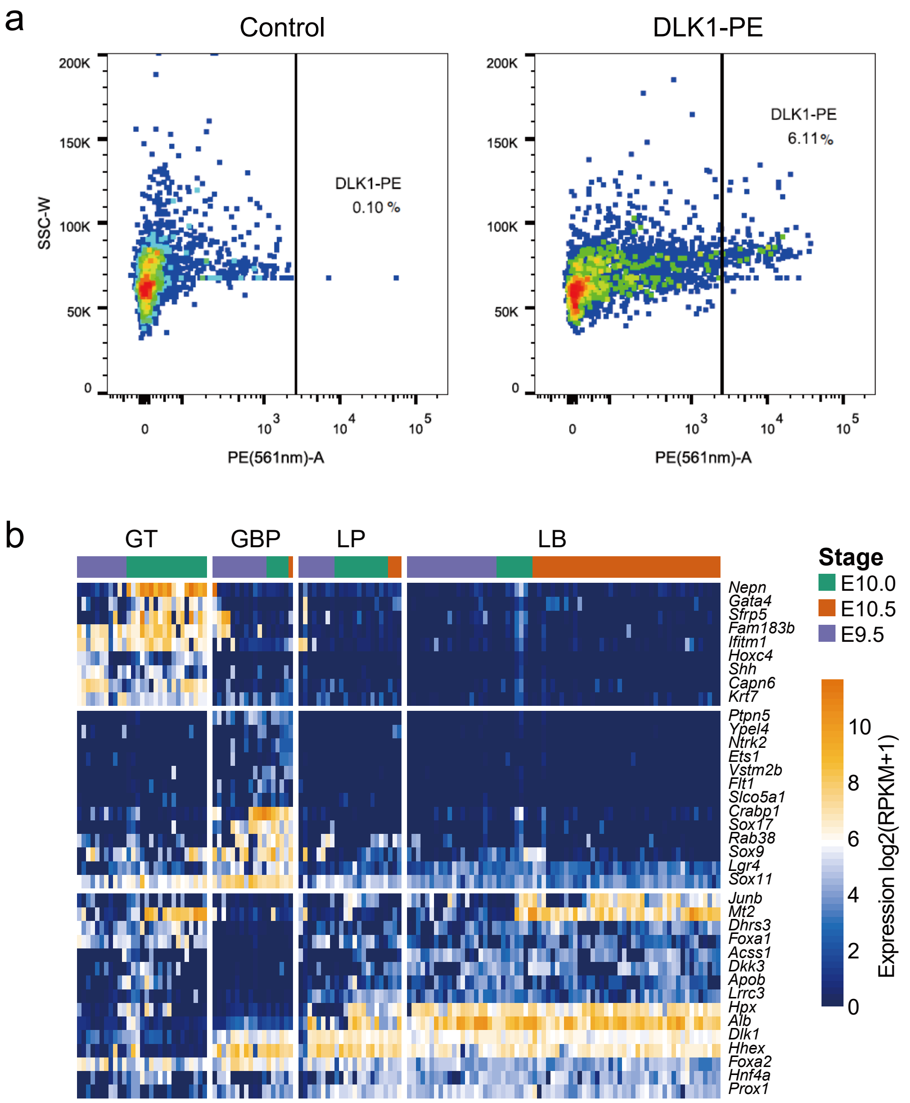

Supplementary Figure 8 | Gene expression in liver primordium and gallbladder primordium. a DLK1 was able to be used as a surface marker to isolate nascent hepatoblasts at E9.5 by FACS. **b** Differentially expressed genes in Gut tube (GT), Gallbladder primordium (GBP), Liver primordium (LP), and Liver bud (LB) during E9.5-E11.5.

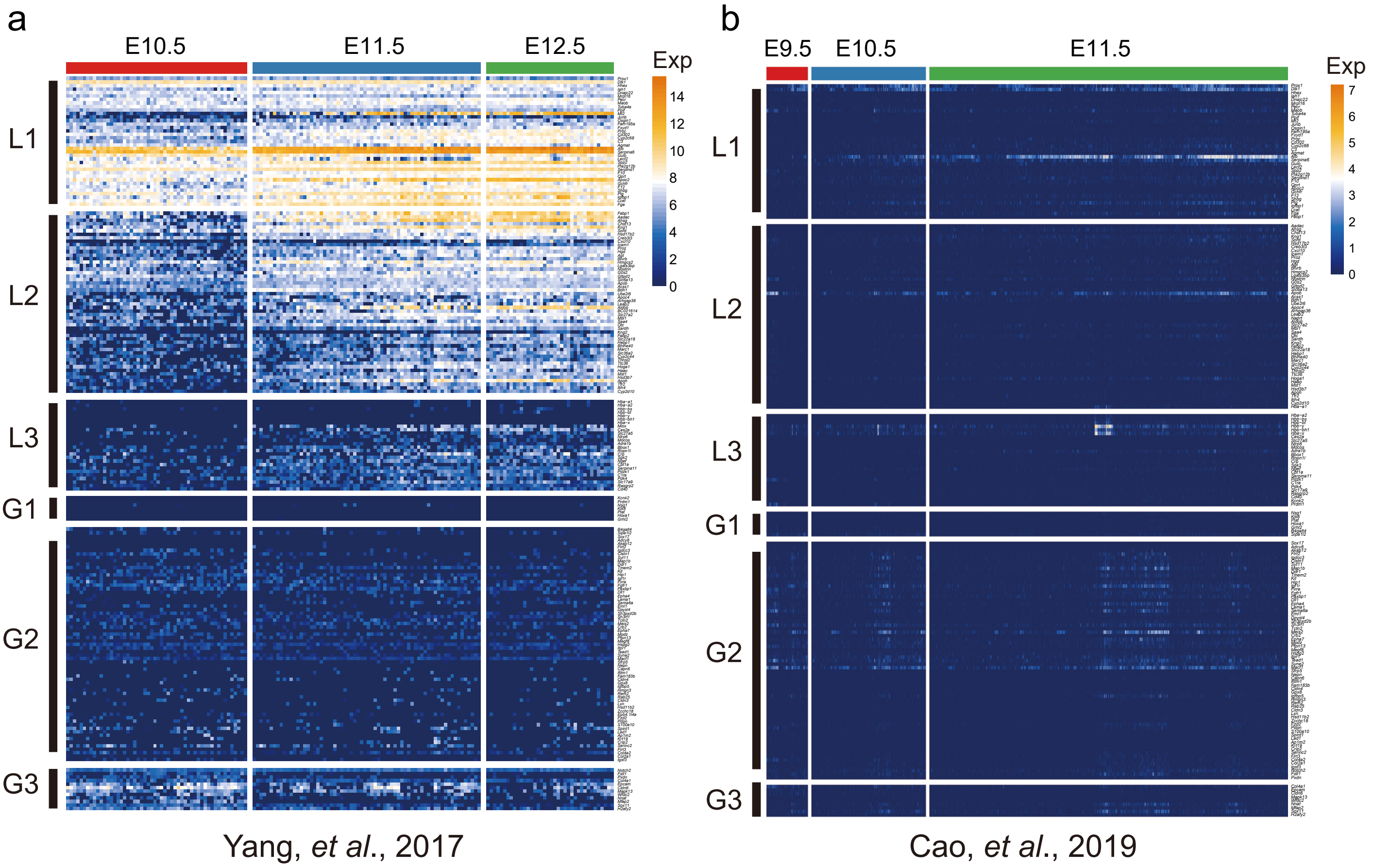

Supplementary Figure 9 | Validation of epithelial-hepatic transition (EHT) genes in two datasets. Heat map illustrated the expression of six groups of EHT genes (L1, L2, L3, G1, G2 and G3) in a full-length mRNA sequencing database (**a**) and a sci-RNA-seq3 database (**b**).

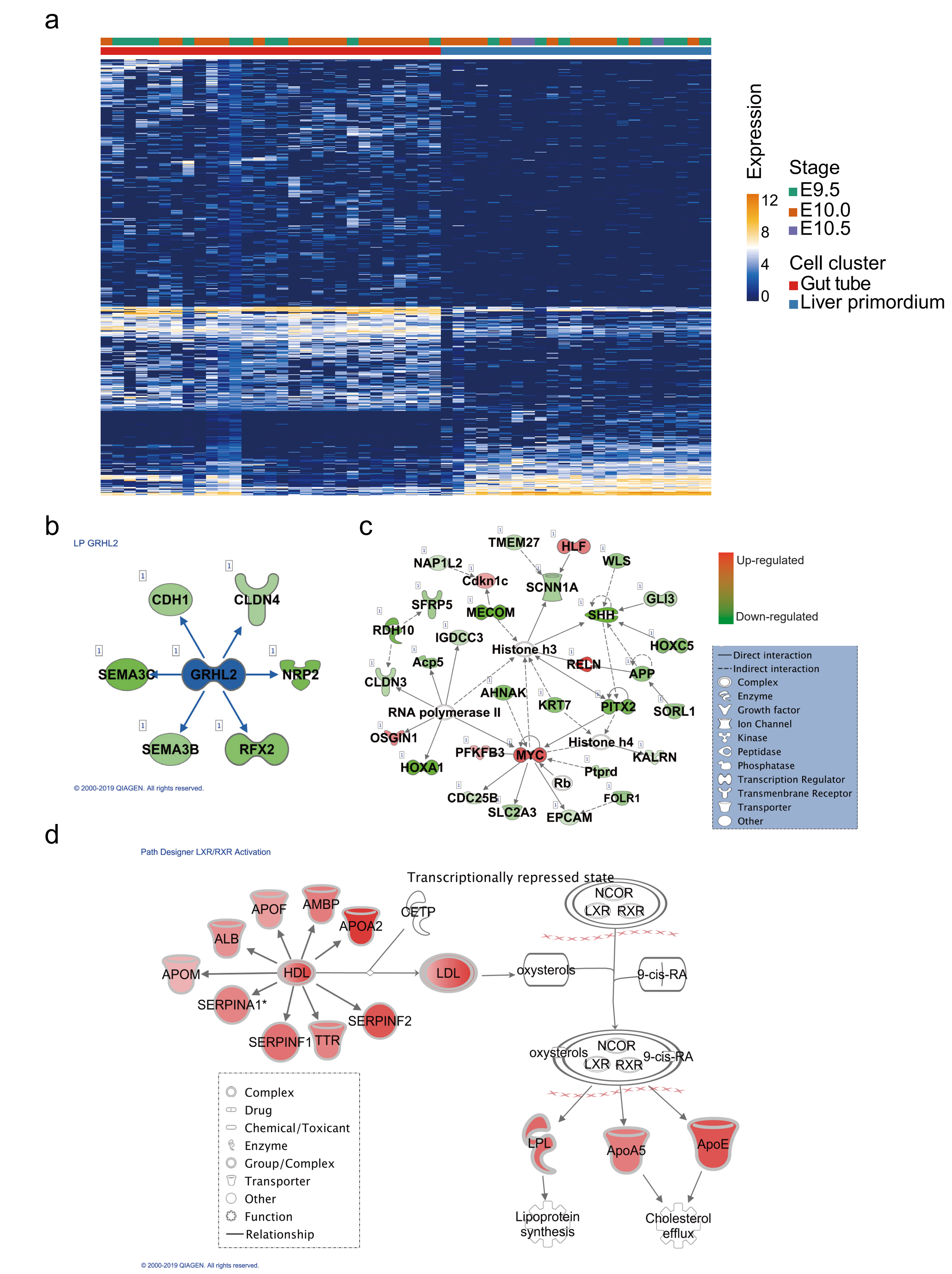

Supplementary Figure 10 | Differentially expressed genes among gut tube and liver primordium. a Heat map illustrated the 548 genes that differentially expressed between the gut tube and liver primordium. **b** *Grhl2* and its downstream targets (*Cdh1*, *Cldn4*, *Sema3c*, *Sema3b*, *Rfx2*, *Nrp2*) were found to be down-regulated in liver primordium, compared with gut tube. **c** The network of differentially expressed genes between gut tube and liver diverticulum, which was enriched in 'Cellular Development', 'Cell Growth and Proliferation', 'Connective Tissue Development and Function', 'Embryonic Development' and 'Organismal Development'. **d** The liver X receptors/retinoid X receptors (LXR/RXR) pathway was significantly up-regulated in liver primordium compared with gut tube.

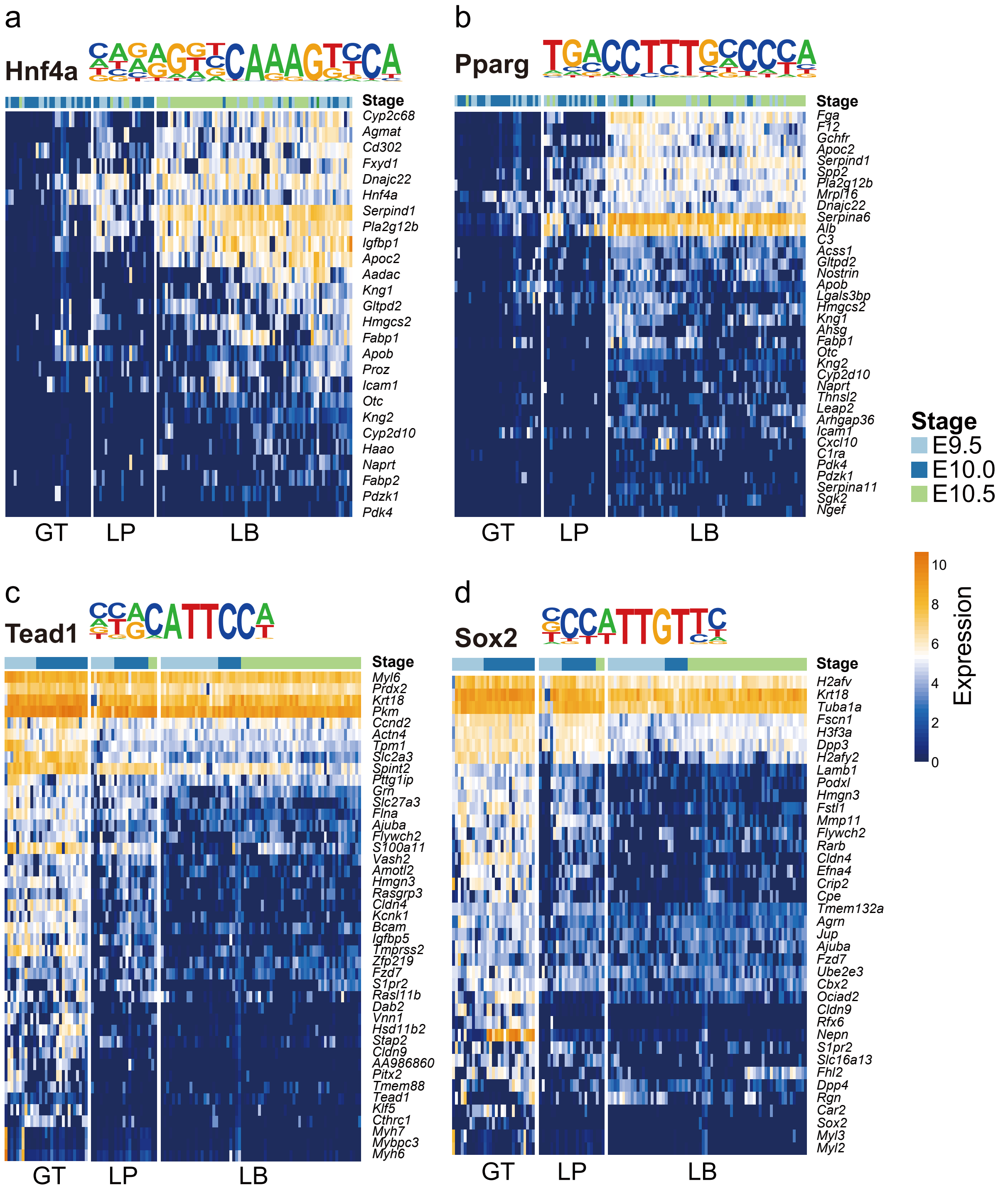

Supplementary Figure 11 | Motif analysis during liver specification. Motif analysis showed targets of *Hnf4a* (**a**) and *Ppagr* (**b**) were up-regulated in liver primordium (LP) and liver bud (LB), while targets of *Tead1* (**c**) and *Sox2* (**d**) were found to be down-regulated. GT, gut tube.

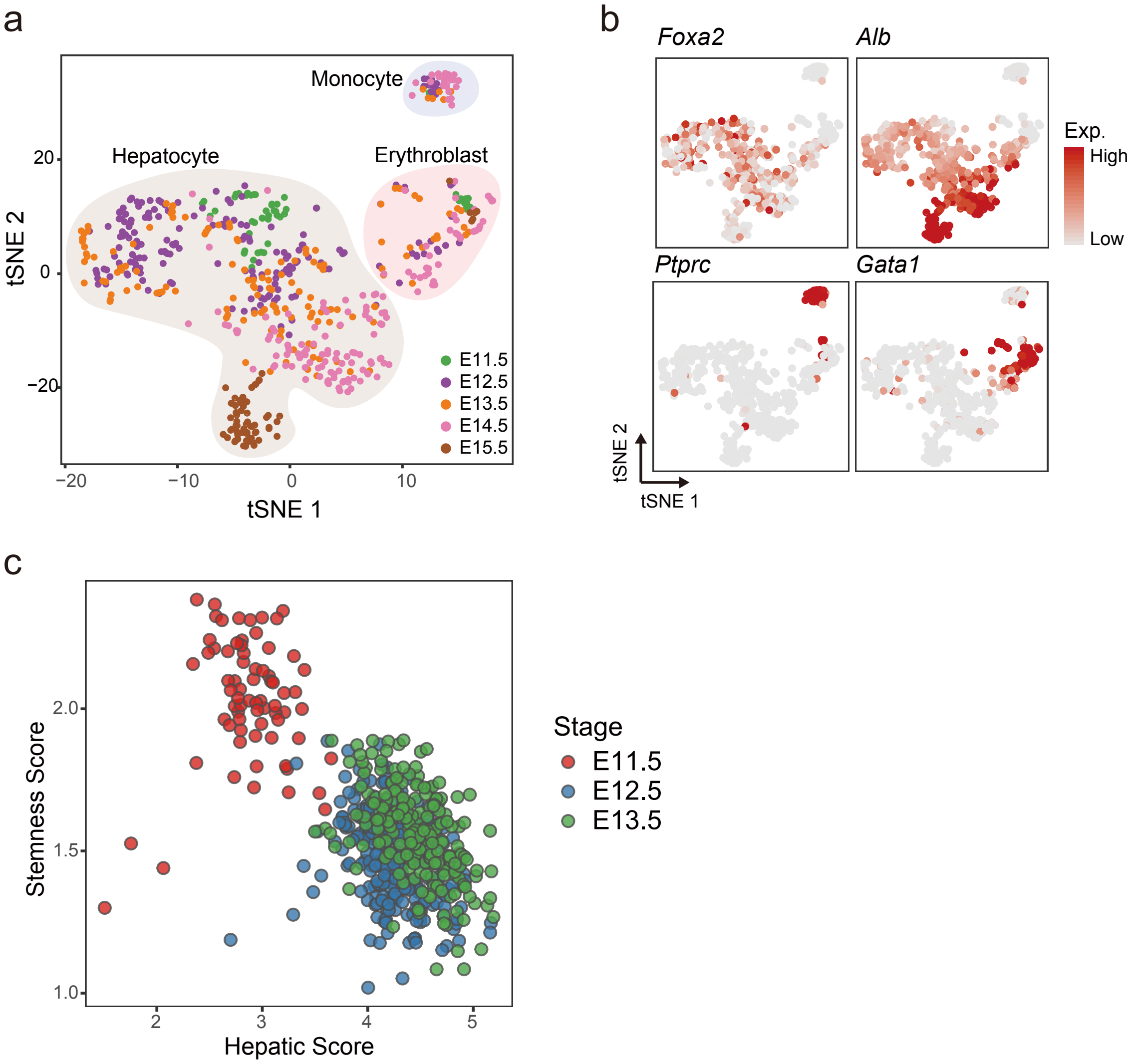

Supplementary Figure 12 | Dynamic gene expression of hepatoblast maturation during E11.5-E15.5. a t-SNE visualization of single-cells from E11.5 to E15.5. **b** The gene expression of specific markers, such as *Foxa2*, *Alb*, *Ptprc*, *Gata1*, was shown. **c** 720 cells generated by MIRALCS method validated that the hepatic score of hepatoblasts/hepatocytes increased, while the stemness score cell decreased during liver maturation.
