# Supplementary material for: Embryonic liver developmental trajectory revealed by single-cell RNA sequencing in the Foxa2eGFP mouse
Source: Commun Biol. 2020 Nov 3;3:642. doi: 10.1038/s42003-020-01364-8 (PMC7642341; doi:10.1038/s42003-020-01364-8)
Supplement: Supplementary file 3 — Description of Additional Supplementary Files [file 42003_2020_1364_MOESM3_ESM.pdf]

Description of Additional Supplementary Files

File Name: Supplementary Data 1
Description: Statistic information of cells.

File Name: Supplementary Data 2
Description: Differentially expressed genes (DEGs) between DE-derived gut and VE-derived gut.

File Name: Supplementary Data 3
Description: Differentially expressed genes (DEGs) between DE-derived gut and PS.

File Name: Supplementary Data 4
Description: Feature genes list for cell state definition.

File Name: Supplementary Data 5
Description: Differentially expressed genes (DEGs) between gallbladder primordium and gut tube.

File Name: Supplementary Data 6
Description: Gene groups of the epithelial-hepatic transition (EHT) dynamic transition.

File Name: Supplementary Data 7
Description: Differentially expressed genes (DEGs) between liver primordium and gut tube.

File Name: Supplementary Data 8
Description: Genes list which are identified with RXRA motif.

File Name: Supplementary Data 9
Description: Dynamic gene expression during hepatoblast development.
