# Supplementary material for: Embryonic liver developmental trajectory revealed by single-cell RNA sequencing in the Foxa2eGFP mouse
Source: Commun Biol. 2020 Nov 3;3:642. doi: 10.1038/s42003-020-01364-8 (PMC7642341; doi:10.1038/s42003-020-01364-8)
Supplement: Supplementary file 13 — Reporting Summary [file 42003_2020_1364_MOESM13_ESM.pdf]

nature research

Corresponding author(s): Xinyuan Fu, Yong Hou

Last updated by author(s): Jun 29, 2020

Reporting Summary

Nature Research wishes to improve the reproducibility of the work that we publish. This form provides structure for consistency and transparency in reporting. For further information on Nature Research policies, see Authors & Referees and the Editorial Policy Checklist.

Statistics

For all statistical analyses, confirm that the following items are present in the figure legend, table legend, main text, or Methods section.

n/a	Confirmed	
☐	☒	The exact sample size (*n*) for each experimental group/condition, given as a discrete number and unit of measurement
☐	☒	A statement on whether measurements were taken from distinct samples or whether the same sample was measured repeatedly
☐	☒	The statistical test(s) used AND whether they are one- or two-sided<br>*Only common tests should be described solely by name; describe more complex techniques in the Methods section.*
☒	☐	A description of all covariates tested
☐	☒	A description of any assumptions or corrections, such as tests of normality and adjustment for multiple comparisons
☐	☒	A full description of the statistical parameters including central tendency (e.g. means) or other basic estimates (e.g. regression coefficient) AND variation (e.g. standard deviation) or associated estimates of uncertainty (e.g. confidence intervals)
☐	☒	For null hypothesis testing, the test statistic (e.g. *F*, *t*, *r*) with confidence intervals, effect sizes, degrees of freedom and *P* value noted<br>*Give P values as exact values whenever suitable.*
☒	☐	For Bayesian analysis, information on the choice of priors and Markov chain Monte Carlo settings
☒	☐	For hierarchical and complex designs, identification of the appropriate level for tests and full reporting of outcomes
☐	☒	Estimates of effect sizes (e.g. Cohen's *d*, Pearson's *r*), indicating how they were calculated

Our web collection on statistics for biologists contains articles on many of the points above.

Software and code

Policy information about availability of computer code

Data collection	The full-length scRNA-Seq and sci-RNA-seq3 processed data were collected from the the NCBI Gene Expression Omnibus repository with accession number GSE90047 and GSE119945 respectively.
Data analysis	SOAPnuke (v1.5.6), TopHat (v2.1.0), Bowtie (v0.12.9.0), Rsubread (v1.16.1), edgeR (v2.6.12), Seurat (v2.1.0), SCDE (v1.99.1), RaceID, Monocle (v2.6.4), Ingenuity Pathway Analysis (INGENUITY®), HOMER (v4.8.3), iTranscriptome (https://www.picb.ac.cn/hanlab/itranscriptome/), DAVID Bioinformatics Resource v.6.8 (https://david.ncifcrf.gov/). The source codes for single-cell bioinformatics analysis n our study can be accessed on GitHub (https://github.com/CellOmics-Yu/Mus_liver_development).

For manuscripts utilizing custom algorithms or software that are central to the research but not yet described in published literature, software must be made available to editors/reviewers. We strongly encourage code deposition in a community repository (e.g. GitHub). See the Nature Research guidelines for submitting code & software for further information.

Data

Policy information about availability of data

All manuscripts must include a data availability statement. This statement should provide the following information, where applicable:

- Accession codes, unique identifiers, or web links for publicly available datasets
- A list of figures that have associated raw data
- A description of any restrictions on data availability

The raw and processed data that generated in this study has been deposited into CNGB Sequence Archive (CNSA: https://db.cngb.org/cnsa) of CNGBdb with accession number CNP0000236. Previously generated drop-based single-cell RNA sequencing data for mouse embryos analyzed in this study can be downloaded from the NCBI Gene Expression Omnibus with accession number GSE119945. Another full-length single-cell RNA-seq for development of mouse embryos hepatocyte is acquired from the NCBI GEO repository with accession number GSE90047. The plasmids in this study are deposited in GenBank (accession number MT936307). In addition, any relevant data upon request is available by contacting the corresponding author (Dr. Yong Hou).

Field-specific reporting

Please select the one below that is the best fit for your research. If you are not sure, read the appropriate sections before making your selection.

☒ Life sciences ☐ Behavioural & social sciences ☐ Ecological, evolutionary & environmental sciences

For a reference copy of the document with all sections, see nature.com/documents/nr-reporting-summary-flat.pdf

Life sciences study design

All studies must disclose on these points even when the disclosure is negative.

Sample size	No explicit calculations were performed to determine sample size. We sorted EGFP-positive embryonic cells from at least three embryos at each stage during E7.5-E15.5.
Data exclusions	Cells with mapping reads < 1 million or mapping rate < 40% were discarded. Cells in 96-wells plates with detected gene number (RPKM > 1) ≥ 6,000 were defined as qualified cells, and the threshold of gene number was set as 4,000 for cells generated by MIRALCS.
Replication	Samples from the hepatic regions from E12.5, E13.5 and E14.5 were repeated by SMART-seq2. In addition to single cells from 96-well plates, libraries from 720 cells from E11.5, E12.5 and E13.5 by MIRALCS (microwell full-length mRNA amplification and library construction system) were generated.
Randomization	Embryos in experiment were randomized and deposited into different wells to perform single-cell RNA-seq.
Blinding	Investigators were blinded to group allocation during data collection and analysis.

Reporting for specific materials, systems and methods

We require information from authors about some types of materials, experimental systems and methods used in many studies. Here, indicate whether each material, system or method listed is relevant to your study. If you are not sure if a list item applies to your research, read the appropriate section before selecting a response.

Materials & experimental systems

n/a	Involved in the study
☐	☒ Antibodies
☒	☐ Eukaryotic cell lines
☒	☐ Palaeontology
☐	☒ Animals and other organisms
☒	☐ Human research participants
☒	☐ Clinical data

Methods

n/a	Involved in the study
☒	☐ ChIP-seq
☐	☒ Flow cytometry
☒	☐ MRI-based neuroimaging

Antibodies

Antibodies used	Antibodies, including FOXA2 (Cell Signaling, #3143); DLK1 (Abcam, ab21682); HNF4A (LSBio, LS-C413074); LZTS1 (Bioss, bs-5705R) were used.
Validation	Antibodies were validated by positive and negative control experiments. These antibodies can be used for immunofluorescence assay and mouse species.

Animals and other organisms

Policy information about studies involving animals; ARRIVE guidelines recommended for reporting animal research

Laboratory animals	Transgenic mouse line FOXA2-EGFP was used. This FOXA2-EGFP mouse line has a C57BL/6 background and was used to collect mouse embryos. The female mice were euthanized normally at 2-3 months.
Wild animals	About 10 wild type mice with a C57BL/6 background were used for the dissection practice and genotyping control.
Field-collected samples	None.
Ethics oversight	All animal procedures were complied with all relevant ethical regulations for animal testing and reseach and performed under the strict instruction by the Institutional Animal Care and Use Committee (IACUC) approval (IACUC approval number R15-0831) of Comparative Medicine of National University of Singapore.

Note that full information on the approval of the study protocol must also be provided in the manuscript.

Flow Cytometry

Plots

Confirm that:

- [x] The axis labels state the marker and fluorochrome used (e.g. CD4-FITC).
- [x] The axis scales are clearly visible. Include numbers along axes only for bottom left plot of group (a 'group' is an analysis of identical markers).
- [x] All plots are contour plots with outliers or pseudocolor plots.
- [x] A numerical value for number of cells or percentage (with statistics) is provided.

Methodology

Sample preparation	Dispase was used to digest tissues from mouse embryos. The single-cell suspension was stained with antibodies before sorting.
Instrument	BD FACSAira III cell sorter
Software	BD FACSDiva 8.0; Flowjo v10
Cell population abundance	The percentage of cell population was labeled in the figure of the manuscript.
Gating strategy	Gating strategy of FSC/SCC was demonstrated in the supplementary figure to exclude doublets. The fluorescence gate was set based on the comparison between experimental group and control group.

- [x] Tick this box to confirm that a figure exemplifying the gating strategy is provided in the Supplementary Information.
